# Acute Febrile Illness Associated with an Emerging Dengue 4 GIIb Variant Causing Epidemic in León, Nicaragua 2022

**DOI:** 10.3390/v17081113

**Published:** 2025-08-13

**Authors:** Omar Zepeda, Edwing C. Cuadra, Daniel O. Espinoza, Yerun Zhu, Hernán Vanegas, Alexis Domeracki, Rodrigo A. Mora-Rodríguez, Anne Piantadosi, Jesse J. Waggoner, Armando J. Matute, Lakshmanane Premkumar, Aravinda M. de Silva, Matthew H. Collins, Megan E. Reller, Filemón Bucardo

**Affiliations:** 1 Centro de Investigación de Enfermedades Tropicales (CIET), Faculty of Microbiology, University of Costa Rica, San José 11501-2060, Costa Rica; zepeomi@gmail.com (O.Z.); rodrigo.morarodriguez@ucr.ac.cr (R.A.M.-R.); 2 Department of Microbiology and Immunology, School of Medicine, The University of North Carolina, Chapel Hill, NC 27559, USA; edcuadra@email.unc.edu (E.C.C.); prem@med.unc.edu (L.P.); aravinda_desilva@med.unc.edu (A.M.d.S.); 3 Division of Infectious Diseases, Department of Medicine, Emory University School of Medicine, Atlanta, GA 30322, USA; daniel.omar.espinoza@emory.edu (D.O.E.);; 4 Facultad de Ciencias Médicas, Universidad Nacional Autónoma de Nicaragua (UNAN-León), León 21000, Nicaragua; 5 Division of Infectious Diseases, Department of Medicine, Duke University School of Medicine, Durham, NC 27710, USA; 6 Research Center on Surgery and Cancer, CICICA, Universidad de Costa Rica, San José 11501-2060, Costa Rica; 7 Department of Pathology and Laboratory Medicine, Emory University School of Medicine, Atlanta, GA 30322, USA; 8 Rollins School of Public Health, Emory University, Atlanta, GA 30322, USA; 9 Hospital Escuela Oscar Danilo Rosales Arguello, Departamento de Medicina Interna, Facultad de Ciencias Médicas, Universidad Nacional Autónoma de Nicaragua (UNAN-León), León 21000, Nicaragua; ajmm201481@gmail.com; 10 Department of Family Medicine, School of Medicine, The University of North Carolina, Chapel Hill, NC 27599, USA

**Keywords:** DENV-4, outbreak, secondary infections, genotype GIIb

## Abstract

Historically, DENV-4 has been rarely associated with epidemics and has been less well-studied than DENV-1 to -3. Epidemic dengue struck several South and Central American countries in 2022, with Nicaragua reporting the highest incidence. In an acute febrile illness (AFI) cohort enrolled from June to September 2022, 58 (34%) of 172 patients had PCR-confirmed dengue, of which 46 (79%) were serotyped as DENV-4. In this cohort, acute dengue, as a proportion of AFI, increased from 8% in June to a peak of 58% in August. Genome sequencing and phylogenetic analysis identified a lineage of DENV-4 Genotype IIb (GIIb) with six amino acid substitutions on the surface-exposed regions of the envelope (E) protein as compared to a reference sequence from 2005. Indeed, two of these mutations appear to be novel and located at G172E or near N174K, an antigenic epitope on domain I. Most (90%, 43/48) DENV-4 patients had pre-existing DENV IgG (secondary dengue), at the acute phase. Secondary dengue was associated with the male sex (prevalence ratio (PR)), 6.88) and being younger than 11 years of age (PR, 8.38). Further analysis showed no association between past Zika exposure and DENV-4 acute illness in older subjects (≥12 years of age). In conclusion, our study describes an epidemic of DENV-4 in León, Nicaragua, associated with a novel lineage of genotype GIIb, which contains two amino acid changes not observed in DENV-4 before 2022.

## 1. Introduction

Symptomatic dengue (dengue fever) is often characterized by fever, myalgia, arthralgia, and retroorbital pain and may progress to hemorrhage, shock syndrome, and death [1]. In 2009, the World Health Organization (WHO) established the following revised criteria to classify dengue disease: (1) dengue without warning signs, (2) dengue with warning signs (abdominal pain, persistent vomiting, fluid accumulation, mucosal bleeding, lethargy, liver enlargement, and increasing hematocrit with decreasing platelets), and (3) severe dengue (dengue with severe plasma leakage, severe bleeding, or organ failure) [2]. Dengue is caused by dengue virus (DENV), an enveloped, single-stranded RNA virus belonging to the *Flaviviridae* family [3]. The mature DENV particle contains the genomic RNA complexed with capsid (C) proteins [4]. This is surrounded by a bilayer lipid membrane anchored with membrane (M) and envelope (E) proteins [5]. Structurally, E protein monomers contain three distinct domains (EDs), designated EDI, EDII, and EDIII [6,7].

There are four antigenically distinct DENV serotypes (DENV-1 to -4), all of which induce both serotype-specific and cross-reactive DENV antibodies [8]. Serotypes are further genetically subdivided into genotypes (G) based on the phylogeny of the gene encoding the E protein. In contrast to DENV-2, which has six genotypes, DENV-1, DENV-3, and DENV-4 each have five genotypes [9,10]. Among DENV-4 genotypes, GI and GII are predominant and appear to have distinct geographic regions [11]. DENV-4 GI circulates exclusively in Southeast Asia, whereas GII was introduced into the Americas around 1980 [11] and has been evolving for the past three decades independently in Southeast Asia as sub-lineage IIa and in the Americas as a sub-lineage IIb [9]. Episodic positive selection observed in the E gene appears to be responsible for lineage diversification [12], but the antigenic differences between lineages of the same DENV-4 genotype remain to be determined [13,14].

Historically, infection with DENV-4 has been less frequently reported and less well-studied than DENV-1 to -3 infections. A review of the global epidemiology of dengue outbreaks between 1990 and 2015 reported DENV-2 as the most frequent (20.7%) cause of outbreaks, followed by DENV-1 (16.7%), DENV-3 (10.9%), and DENV-4 (4.0%) [15]. Co-circulation of more than one DENV serotype during epidemics is common; however, those with DENV-4 are rarely reported [15]. Over the past 50 years, clinical trials have evaluated a variety of therapeutic strategies with limited success [16]. While supportive care and prudent hydration therapy have shown improvement in patients with dengue, there is currently no specific treatment for dengue [17]. In this context, epidemiological surveillance studies are essential, as they allow for the identification of transmission patterns, early detection of outbreaks, and guidance in the design of new evidence-based therapeutic and preventive interventions.

In this study, we describe a DENV-4 epidemic detected by acute febrile illness (AFI) surveillance in western Nicaragua in 2022 and examine the clinical, epidemiological, serological, and molecular characteristics of this outbreak.

## 2. Materials and Methods

### 2.1. Nicaraguan Acute Febrile Illness (AFI) Cohort and Samples

Our hospital and community surveillance cohort for AFI, established in León, western Nicaragua, in 2018, detected an increase in the proportion of AFI cases attributable to acute dengue in 2022. The current study includes adult and pediatric patients recruited between 1 June and 30 September 2022, at the emergency department of the main local hospital and two community clinics (further details in Supplement S1). Patients provided acute- and convalescent-phase samples with and without coagulant. Within 3 h of venipuncture, blood parameters were determined, sera aliquoted, and specimens stored at −80 °C. PCR for dengue, chikungunya, and Zika was performed on site in Nicaragua. Two separate aliquots of each serum sample, not previously thawed, were shipped to Emory University and the University of North Carolina at Chapel Hill on dry ice for dengue and Zika serology, virus isolation, and sequencing.

### 2.2. Ethics Statement

Written informed consent was obtained from participants or their guardians (for patients ≤ 18 years of age), and written assent was obtained from patients aged 12–17 years. The study protocol was reviewed and approved by the institutional review boards of UNAN-León (FWA00004523/IRB00003342) and Duke University (Pro00014461). The research on de-identified specimens was declared exempt from IRB review by Emory University.

### 2.3. Blood Parameters Determination

Blood samples were collected in ethylenediaminetetraacetic acid (EDTA) Vacutainer tubes and the hematological profile including white blood cell (WBC) count, differential leukocyte count (neutrophils, lymphocytes, monocytes, eosinophils, and basophils), platelet count, hemoglobin concentration, hematocrit, red blood cell count, and mean corpuscular volume (MCV) was determined using an automated blood analyzer (Edan H30 Pro, EDAN, Shenzhen, China). All readings were performed within 3 h of sample collection.

### 2.4. Arbovirus PCR Screening and Dengue Serotyping

In Nicaragua, RNA was purified from 140 µL of serum using the QIAamp Viral RNA Mini Kit (QIAmp, Hilden, Germany). Viral RNA reverse transcription (RT) and quantitative polymerase chain reaction (qPCR) were performed in a CFX96 real-time PCR detection system (Bio-Rad, Hercules, California, USA), following CDC’s Trioplex assay [18], with minor modifications. All RT-qPCR positive and a subset of randomly selected DENV-negative samples were re-tested at Emory University to define serotypes and to increase the rigor of molecular testing results (further details in Supplement S1). Procedures for the triplex (DENV, ZIKV, and CHIKV) PCR and DENV serotyping RT-qPCR assays are described elsewhere [19,20]. RT-qPCR and NS1 antigen detection have shown good correlations in samples collected during acute dengue; indeed, RT-qPCR is more sensitive in later stages [21].

### 2.5. Dengue Genome Sequencing and Phylogenetic Analysis

A subset (*n* = 16) of samples with Ct values ≤ 32 were selected for full genome sequencing. Total nucleic acid was treated with dsDNase (ArcticZymes, Tromso, Norway). RNA was converted into cDNA using random hexamer primers and SuperScript III RT (Fisher/Invitrogen) for first-strand synthesis, and New England Biolabs reagents for second-strand synthesis without amplification. Libraries were fragmented, dual-indexed, and amplified with 16 PCR cycles using the Nextera XT DNA Library Prep kit (Illumina). Libraries were quantified using the KAPA universal complete kit (Roche), pooled to equimolar concentration, and sequenced on a MiSeq with paired-end 150 bp reads (Illumina), with a mean of 935,280 reads per sample (range 502,300–1,560,818) (further details in Supplement S1).

Reads underwent reference-based DENV genome assembly using, as the DENV-4 reference, the EU854296 genome and viral-ngs version 2.0.21.3 (https://github.com/broadinstitute/viral-ngs, accessed on 1 March 2023). Dengue serotypes and genotypes were determined using the Dengue Virus Typing Tool [22]. Sequences of the Nicaraguan 2022 isolates were then aligned with 280 reference sequences, which were selected as belonging to DENV-4 genotype II, including complete genome sequences (>94% coverage) with known dates and locations of sampling. A maximum-likelihood tree was constructed from the full coding region using a GTR+F+I+G4 model (best fit) in IQ-TREE version 2.0 [23]. Trees were visualized using iTOL [24]. A pairwise distance (PD) calculation was performed to determine the degree of homology between the Envelope (E) protein from Nicaraguan 2022 isolates and the E protein from all known reference DENV-4 strains, including vaccine strains. DENV-4 phylogenetic analysis of the E gene from the Nicaraguan strain, recent and remote strains circulating in the Caribbean basin, and reference strains representative of different DENV-4 genotypes and lineages was performed using MEGA 11.0.13, and the tree was constructed using the neighbor-joining and Kimura two-parameter methods.

### 2.6. Antigen Capture IgG ELISA

Binding IgG to DENV was measured by an antigen capture ELISA as previously described [25] (further details in Supplement S1). ELISA data were reported as OD values that were the average of technical replicates. The cutoff for positivity in each plate was 0.1 plus the average OD of the N (NCs) plus 3 standard deviations.

### 2.7. ZIKV EDIII ELISA

High-binding microtiter wells (Greiner Bio-One, Cat. No. 655061, Kremsmünster, Austria) were coated with 50 μL of streptavidin (Invitrogen, Cat. No. 434302, Waltham, MA, USA) at 4 μg/mL in tris-buffered saline (TBS, pH 7.4) for 1 h at 37 °C. An in-house site-specifically biotinylated ZIKV EDIII antigeb was captured (50 µL/well at 2 μg/mL in TBS) in the wells as described before [26]. A total of 50 μL of heat-inactivated serum diluted in TBS (1:20) was tested in duplicate. A 3-fold serially diluted (2.5 μg/mL) Zika monoclonal (ZKA190) was used as a positive control in every assay plate. Goat anti-human IgG alkaline phosphatase conjugate was used as the detection antibody (Sigma-Aldrich, Cat. No: A9544, Burlington, MA, USA). Optical density (OD) was recorded at 405 nm using a SpectraMax ELISA plate reader. The concentration of Zika EDIII IgG antibodies in each sample was determined by extrapolating the OD reading in a standard curve prepared in GraphPad 10.0.0 and reported as binding antibody units (1 BAU = 1 ng/mL). Serostatus was determined as follows: positive > 18.5 BAU, negative < 17.5 BAU, and indeterminate 17.5–18.5 BAU.

### 2.8. Laboratory Definitions

The acute phase sample was collected at the unit attending febrile illness from patients with documented fever (≥38 °C) in the past 48 h. Acute dengue cases were rigorously confirmed by detection of viral RNA using pan-DENV RT-qPCR, followed by serotype-specific RT-qPCR (DENV-1 to -4). Paired acute and convalescent sera were evaluated for anti-DENV IgG to support serological confirmation. Infections were classified as primary or secondary based on the absence or presence of dengue-specific IgG in acute-phase serum, respectively. Cross-sectional dengue and Zika seroprevalences were performed in the acute samples, and they were defined based on the presence or absence of DENV IgG (DENV positive) and Zika EDIII IgG (Zika Positive).

### 2.9. Statistical Analysis

We compared dengue IgG seroprevalence in those with and without acute dengue in relation to sociodemographic, epidemiological, and clinical characteristics by using the Chi-square test or Fisher’s exact test for categorical variables and the *t*-test or Kruskal–Wallis test for continuous variables. We used a conservative Bonferroni correction of *p* values and confidence intervals where applicable to indicate statistical significance [27]. Hematological alterations in patients with and without dengue were compared using geometric means (GMs) or arithmetic means (for eosinophils only). Group comparisons were performed using one-way analysis of variance (ANOVA) on log-transformed values, followed by Tukey’s Honest Significant Difference (HSD) and an unpaired Student’s *t*-test for comparisons. SPSS, STATA, version 11 (STATACorp, College Station, TX, USA), R (R version 4.2.2), and GraphPad Prism 8 were used for statistical analyses and plotting.

## 3. Results

### 3.1. AFI Surveillance Detected a Sharp Increase in DENV-4

**Figure 1:**
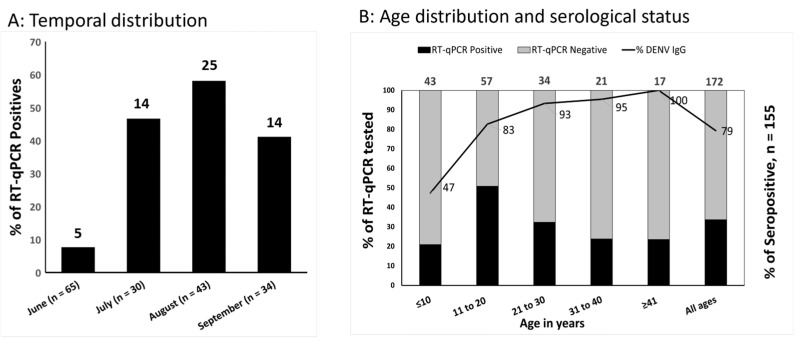
(**A**) Temporal distribution of acute dengue (RT-qPCR-positive) versus other febrile illness (RT-qPCR-negative) between June and September 2022 in León, Nicaragua. (**B**) Age distribution and serological status of individuals with acute dengue vs. other febrile illness. The primary *Y*-axis’ stacked bars represents the proportion of patients who were dengue RT-qPCR-positive and -negative in each age group. In the secondary Y-axis, the solid black line across the bars represents the proportion of DENV IgG-seropositive patients by age. In both figures, the total number of tested patients is provided at the top of the bar for each category.

RT-qPCR screening for DENV, Zika, and Chikungunya found that the proportion of AFI attributable to acute dengue increased from 8% in June to 58% in August (Figure 1A); overall, 58 (33.7%) of 172 patients enrolled with AFI during the study period had acute DENV and none had Zika or Chikungunya virus. Of the 48 that could be serotyped by conventional RT-qPCR, 46 (95.8%) were DENV-4 and 2 (4.1%) were DENV-1. No DENV co-infections were identified. DENV-4 (*n* = 15) and DENV-1 (*n* = 2) were successfully isolated by cell culture from acute serum (Supplement S2). The infecting serotype was not confirmed in 5 of the 48 samples tested at Emory, due to either high Ct values or differences in assay sensitivities. There was a good correlation between the Ct values of the RT-qPCRs performed at UNAN-León and Emory (R^2^, 0.998).

### 3.2. Acute Dengue Was Associated with Age, Sex, and Hospital Admission

Acute dengue occurred more frequently in males (PR: 1.67; 95%CI: 1.09–2.56) and those 12 to 18 years of age (PR: 2.38; 95%CI: 1.31–4.32, Table 1, Figure 1B). Those with acute dengue, compared with other AFIs, were nearly twice as likely to be admitted to hospital (PR: 1.99; 95%CI: 1.34–2.97), a statistically significant finding. Patients clinically classified as suspected COVID-19 (not laboratory-confirmed) were more likely to have acute dengue (PR: 1.71; 95%CI: 1.10–2.64). AFI patients reporting prior dengue vaccination (2 of 5) had confirmed acute DENV-4 infection (Table 1). TAK-003 (QDENGA) was being used in Leόn, Nicaragua, as part of a clinical trial during the time frame of the current outbreak.

### 3.3. Clinical and Hematological Characteristics

The clinical characteristics associated with PCR-confirmed acute dengue versus other AFIs included headache (PR: 2.71; 95%CI: 1.17–6.26), rash (PR: 1.93; 95%CI: 1.21–3.06), joint pain (PR: 1.72; 95%CI: 1.08–2.74), and retroorbital pain (PR: 1.56; 95%CI: 1.03–2.37), none of which were statistically significant after adjustment but all showed a trend toward association (Supplement S3). In contrast, respiratory symptoms (e.g., sore throat, rhinorrhea, and cough) were less frequent in those with RT-qPCR-confirmed acute dengue (Supplement S3). Those with acute dengue compared with other AFIs had lower mean white blood cell counts (5.82 vs. 9.00 × 10^3^/µL), including lower proportions of eosinophils (1.56% vs. 3.63%) and lower platelet counts (223 vs. 258 × 10^3^/µL). Bonferroni correction showed that white blood cells and platelets were highly associated with acute dengue virus infection (Table 2).

### 3.4. Most DENV-4 Infections Constitute Secondary Acute Dengue

To investigate whether acute DENV-4 cases occurred in DENV-naïve individuals or individuals with past DENV infection, all available (*n* = 155) acute-phase sera were tested for DENV IgG-binding Ab. A total of 123 (79%) of 155 patients from this study were DENV IgG-seropositive at presentation with AFI. In total, 50% of children ≤11 years of age were DENV IgG-seropositive, as well as 87% of subjects 12–18 years of age, 89% of those aged 19–29, and >95% of those older than 30 years (Table 3). A similar proportion of males (81%) and females (78%) were DENV IgG-seropositive at enrollment. Most acute dengue infections were secondary infections; thus, a higher proportion of IgG-positive patients had acute dengue infection confirmed by RT-qPCR compared to seronegative patients in general (35% vs. 16%, PR: 2.24; 99%CI: 0.74–6.75). Furthermore, acute secondary dengue occurred disproportionally in children ≤11 years (PR: 8.38; 99%CI: 0.61–114.73) and in males (PR: 6.88; 99%CI: 0.56–84.29) (Table 3).

### 3.5. Zika Serostatus

Zika EDIII IgG was detected in 37% of participants. No association between Zika seropositivity and acute dengue was observed between sexes and between age groups, except for a trend toward association in children ≤11 years of age (PR: 4.20; 99%CI: 1.01–17.41) (Table 3).

### 3.6. Hematological Alterations Were Most Pronounced in Acute Secondary Dengue

**Figure 2:**
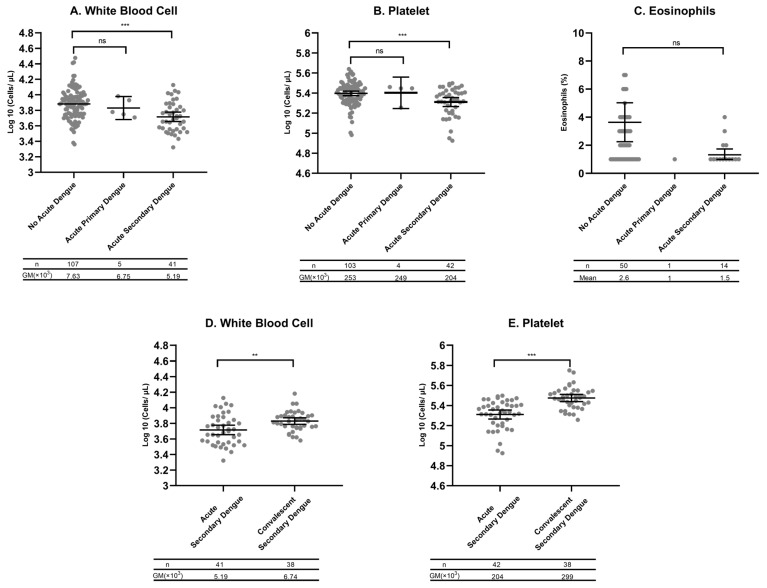
GMs and mean plots with 95% confidence intervals (CI). (**A**) White blood cell counts (WBCs), (**B**) platelet counts, and (**C**) eosinophil percents among no acute dengue (dengue RT-qPCR-negative), acute primary dengue (dengue IgG-negative and RT-qPCR-positive), and acute secondary dengue (dengue IgG-positive and RT-qPCR-positive). To account for data skewness, all values were log-transformed prior to statistical analyses. GMs and their 95% CIs were estimated on the logarithmic scale and subsequently back-transformed for visualization. The table below each figure shows the *n* value for each category and the GM. (**D**) WBCs and (**E**) platelet counts in blood collected from those with secondary dengue during the acute and convalescence phases. Ns: ns stands not significant; ** *p* < 0.001; *** *p* < 0.0001 according to Tukey’s Honest Significant Difference (HSD) post hoc test and an unpaired Student’s *t*-test comparing differences between groups. WBC values are expressed as cells ×10^3^/μL, platelet counts as cells ×10^3^/μL, and eosinophil counts/100 cells.

Further analysis shows that the GMs of WBCs and platelet counts were significantly lower in secondary dengue compared to non-dengue AFIs (WBC 5.19 vs. 7.63, *p* < 0.0001; platelets 204 vs. 253, *p* < 0.0001, Figure 2A,B). In contrast, eosinophil counts did not significantly differ between secondary dengue and non-dengue AFIs (Figure 2C). As expected, WBCs and platelets counts significantly increased (normalized) in blood samples collected at convalescence in subjects with secondary acute dengue, compared to cells counts performed in acute samples from the same subjects (WBC 5.19 vs. 6.74, *p* < 0.001; platelet 204 vs. 299, *p* < 0.0001, Figure 2D,E).

### 3.7. Agreement Between Paired IgG Serology and RT-qPCR

**Figure 3:**
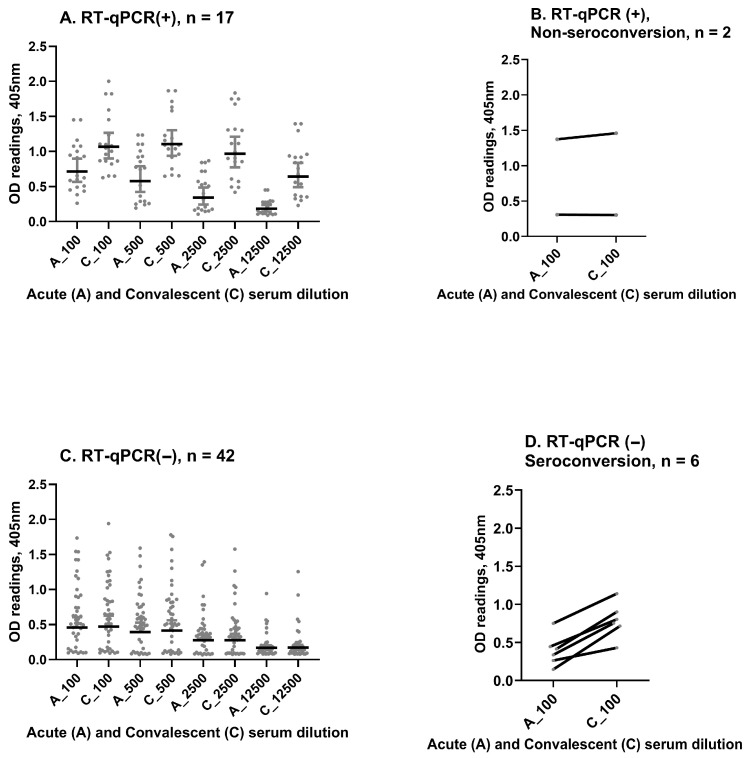
(**A**) Paired IgG serology in available samples (*n* = 67), dengue IgG optical density (OD) increases from acute to convalescent sera at different dilutions in patients with acute dengue confirmed by RT-qPCR. (**B**) Discrepancies observed in 2 RT-qPCR-positive patients without a rise in dengue IgG OD between acute and convalescent sera. (**C**) Dengue IgG OD remains stable in acute and convalescent sera across different dilutions in patients who were dengue RT-qPCR-negative. (**D**) Discrepancies observed in 6 dengue RT-qPCR-negative patients that had rising dengue IgG titers (by OD increase). OD readings are given on the Y-axis, and paired dilutions are shown on the X-axis.

To investigate the concordance between dengue RT-qPCR and paired IgG serology, a total of 67 paired acute and convalescent sera were examined (Figure 3) blindly to RT-qPCR results. In this subset, 17 (89.5%) of 19 dengue RT-qPCR-positive patients had paired IgG serology consistent with acute dengue (Figure 3A,B). In contrast, 42 (87.5%) of 48 dengue RT-qPCR-negative patients showed no increase in IgG in the convalescent sample, consistent with AFI associated with other etiologies (Figure 3C). The six discordant samples were taken within 4 days of the onset of illness and were acute-phase RT-qPCR-negative, yet showed increasing optical densities (ODs) (Figure 3D) between the acute and convalescent timepoints, potentially indicative of recent/current dengue infection. Overall, we observed substantial agreement between dengue RT-qPCR and paired IgG serology (kappa = 0.724, 95%CI: 0.547–0.901). A sensitivity analysis showed that discordant results did not affect the association previously observed (Supplement S4).

### 3.8. The Infecting DENV-4 Belongs to Genotype II, Subcluster IIb

**Figure 4:**
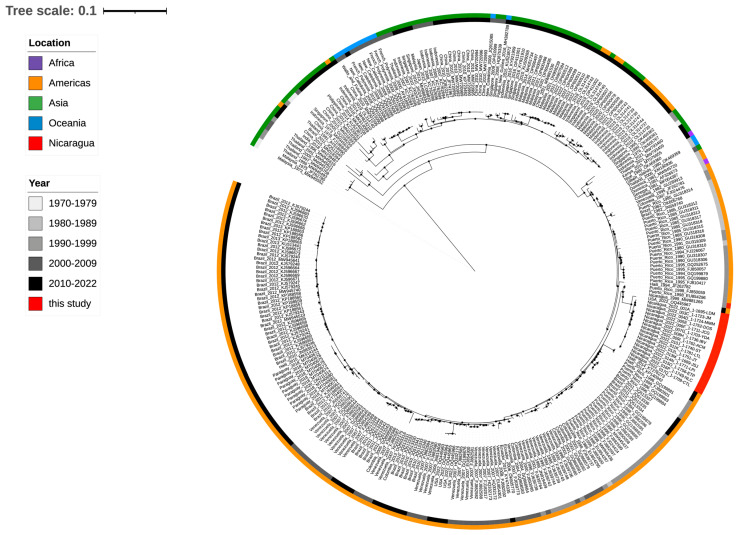
Whole-genome phylogenetic analysis of 16 DENV genome sequences from Nicaragua 2022 (GenBank PP128470–PP128485) and single-genome sequences from Florida, USA, Chiapas, Mexico, and El Salvador circulating between 2021 and 2022. Reference sequences from DENV-4 circulating in endemic countries since 1973 are also included. Color code indicates the continent of the sequence’s origin, and gray color indicates the year of circulation. Red is indicative of Nicaraguan 2022 strains.

**Figure 5:**
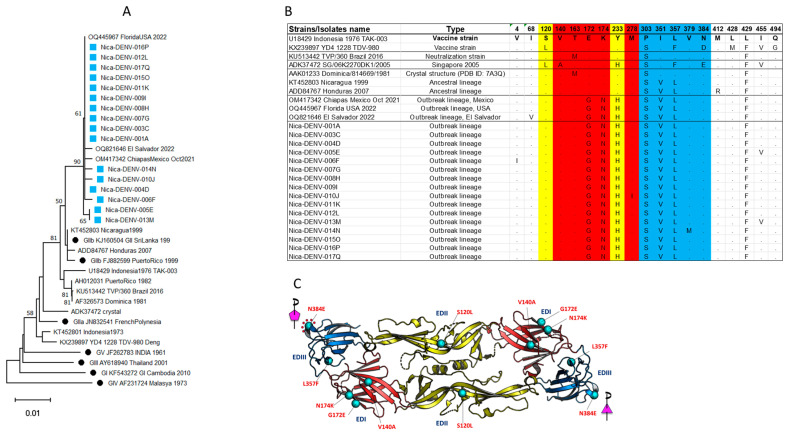
(**A**) Phylogenetic analysis of the amino acid sequences of the E protein from Nicaraguan isolates in 2022 (light blue square) and Florida, Mexico, and El Salvador isolates, including reference sequences from all known DENV-4 genotypes and GII sub-lineages (black dots) besides the E insert in Dengue vaccines. (**B**) Alignment of amino acid sequences from the current study and relevant references emphasizing key amino acid of the antigenic sites of the E protein (full alignment is shown in Supplement S5). The mutations G172E, N174K, and H233Y were not present in the IIb lineage circulating before 2022 in Meso- and South America. E domains are color coded, domain I is red, domain II is yellow, and domain III is blue. (**C**) The amino acid changes in the Nicaraguan 2022 DENV-4 isolates were mapped onto the most recent DENV-4 crystal structure available (SG/06K2270DK1/2005 (PDB ID: 7A3Q).

Phylogenetic analysis of full-length DENV-4 sequences from our cohort (GenBank PP128470–PP128486) and full-length reference sequences from GenBank showed that all DENV-4 sequences isolated in Nicaragua clustered together and belonged to GII, sub-lineage “IIb” (Figure 4). They were most closely related to another full DENV-4 sequence from a case in Florida reported in late 2022. Additional reference sequences were identified for the E gene only, and phylogenetic analysis linked two other recent samples (Chiapas Mexico in 2021 and El Salvador in 2022, Figure 5A). The Nicaraguan outbreak sequences were part of a lineage that was detected in Honduras in 2007 (E gene only, Figure 5A), and prior to that in Nicaragua in 1999 (Figure 4 and Figure 5). Notably, they were distinct from DENV-4 GII viruses circulating before 2022 in Central and South America and from other DENV-4 viruses found in travelers from the Caribbean to Florida USA before 2022 (Figure 4).

### 3.9. Novel Mutations Were Observed in the E Protein of the Nicaraguan 2022 Isolates

Comparison of the E protein from the Nicaraguan 2022 isolates and reference strains demonstrated two mutations that were not detected before 2022: G172E and N174K (Figure 5B). Thus, the outbreak DENV-4 sequences and those from Florida, USA, Chiapas, Mexico, and El Salvador all had two novel amino acid substitutions in E protein not observed in any of the DENV-4 E sequences reported in GenBank before 2022 (Figure 5B), or in the E sequence of the DENV-4 GII strain that is commonly used for the detection of neutralization antibody (strain TVP/360_Brazil_2016) [28]. The novel E sequences from Nicaraguan 2022 DENV-4 were also distinct compared to DENV-4 circulating in Southeast Asia in the 1970s and 1980s, some of which represent prototype strains for leading dengue vaccines (strain Indonesia 1976 and strain 1228, Figure 5B).

### 3.10. Amino Acid Changes Occurred on Domains I, II and III

To further explore the possible impact of these mutations on antigenicity, we analyzed the E substitutions located on the surface-exposed regions of the 2022 DENV4 outbreak strain by using the available crystal structure of DENV-4 (Figure 5C). Comparison of the amino acid changes in the DENV-4 Nicaraguan 2022 isolate to a Singapore DENV-4 2005 isolate (SG/06K2270DK1/2005, PDB ID: 7A3Q, GenBank ADK37472) showed six amino acid changes distributed across EDI, EDII, and EDIII domains (Figure 5C). These included three E Domain I changes (V140A, G172E, and N174K), one Domain II change (S120L), and two Domain III changes (L357F and N384E). None of these changes fell within the epitopes previously defined by the DENV cross-reactive antibody-dependent enhancement [10]. However, the substitution of glutamate by asparagine at position 384 (N384E) sits at the five-fold EDIII interface of the whole virion, which may impact the virus ‘s stability or binding of the antibody targeting around this region.

## 4. Discussion

We report the emergence of DENV-4 as a major cause of AFI in León, Western Nicaragua, in the second half of 2022. The clinical and epidemiological profile of the AFI outbreak was characteristic of secondary acute dengue illness associated with hematological alterations suggestive of increased severity. DENV-4 acute illness has historically been less frequently reported in Nicaragua, but a recent report suggests that severe disease was more prevalent among secondary DENV-2 and DENV-4 cases [29].

Full genome and phylogenetic analyses confirmed that the emerging DENV-4 belonged to genotype II of sub-lineage B. Furthermore, sequenced DENV-4 strains from Nicaragua 2022 and sequences from DENV-4 isolates from Florida, USA, El Salvador, and Chiapas, Mexico, in 2022 carried several amino acid substitutions in the E protein not observed in DENV-4 strains circulating before 2022 in Meso- and South America. The unexpected high proportion of AFIs attributable to acute DENV-4 (33.7%) occurred disproportionately in children, males, and those with DENV IgG consistent with secondary dengue. The emergence of DENV-4 reported in the current study occurred in the context of one of the largest dengue epidemics in Mesoamerica (cumulative incidence 124.69 cases per 100,000 inhabitants) [30] with 10 countries reporting DENV-4 [30] circulation. The presence of a DENV strain with different genetic makeup and fitness might be just one factor contributing to the epidemic, with host prior infections, vector, and environmental factors also important.

Compared with other DENV serotypes, DENV-4 has been rarely reported in AFI studies [31,32,33]. Circulation of DENV-4 in Nicaragua was first documented in the early 1990s and then in 2005 (limited) [31]. Therefore, it is expected that pre-existing immunity in the current cohort with acute DENV-4 would be cross-reactive (against DENV-3, DENV-2, and DENV-1). Indeed, 79.3% of patients from this study were DENV IgG-seropositive at enrollment. Notably, AFI due to DENV-4 was common in those with pre-existing DENV immunity, suggesting the lack of protective population immunity to the emerging DENV-4 variant in western Nicaragua. These findings might correlate with recent observations showing that protection against symptomatic dengue infection by neutralizing antibody varies by infection history and infecting serotype [34].

These findings align with previous observations showing that in flavivirus-naive individuals, infection with DENV-1 and DENV-3 is more frequently observed than with DENV-2 and with DENV-4 [15]. Reports of primary DENV-4 outbreaks are very rare, for instance, between 1990 and 2015, only 4% of the primary dengue outbreaks were associated with DENV-4. Whether antibody-dependent enhancement is required to develop clinical manifestations after DENV-4 infection remains to be elucidated. Low herd immunity to this DENV-4 GII variant coupled with pre-existing DENV-2 and DENV-3 antibody but not Zika antibody may have contributed to the sharp increase in symptomatic DENV-4 infection in Leon, Nicaragua. The finding that hospital admission was significantly higher (PR = 1.99) in patients with dengue-associated AFIs is consistent with recognition of dengue warning signs more frequently observed in secondary infection.

The dominant genetic variant reported here belonged to DENV-4 genotype II, specifically to the sub-lineage IIb that has been evolving independently in the Americas [11]. The E protein from all sequences in this study contained two amino acid changes (G172E and N174K), which were not previously observed in the sub-lineage IIb circulating in this region, nor in DENV-4 of sub-lineage IIa, which has been circulating in the Caribbean basin since the introduction of DENV-4 in 1981 [9,11]. This is a notable finding since prior studies suggest a potential role of these sites in neutralization susceptibility and virus escape [10,35]. For example, DENV-4 neutralization escape variants generated against the chimpanzee monoclonal antibody 5H2, which recognizes EDI, most commonly harbor a single mutation at position 174 (Lys174Glu) [35]. Structural analysis has shown that Lys174 of DENV-4 E is conserved among the other DENV serotypes [35]. Other studies mapping the human memory B cell and serum neutralizing antibody responses to DENV-4 following natural infection or vaccination have shown that amino acid positions in or near the EDI/II hinge region (K51, V53, K124, L135, K200, and K234) are critical for type-specific neutralization [36]. Whether the changes in amino acid observed in the current study have implications for DENV-4 biology or neutralization warrants further investigation. Recent studies have shown that a small number of changes in amino acid within the E protein have disproportionate impacts on virus biology [10]. Future studies assessing the impact of these mutations on viral fitness, immune escape, or vaccine efficacy will be needed.

Molecular epidemiological studies from Peru, Paraguay, Brazil, and Indonesia have shown that genetic changes are associated with the reemergence or introduction of DENV-4 [37,38,39,40]. The sharp increase in DENV-4 as a cause of AFI observed in this and other studies might reflect a complex interaction between viral genetics and population dynamics of type-specific immunity, as suggested previously [32].

The number of dengue-vaccinated subjects with acute DENV-4 in this study was limited. However, it has been previously reported that the efficacy of the CYD tetravalent DENV vaccine against DENV4, in seronegative children, decreased significantly with increasing amino acid distance from the DENV4 vaccine insert [41]. Thus, further investigations are needed to define vaccine efficacy at sub-lineage levels.

Limitations of the current study include a lack of follow-up during hospital admission, which precludes detailed descriptions of dengue acute illness severity. For instance, no severity scores, complications, and lengths of stay were recorded. Furthermore, given the small sample size, our correction for multiple comparisons may have been overly conservative, potentially limiting the detection of statistically significant associations. We include results without the Bonferroni correction in Supplement S6 suggesting that stronger associations might be identified in future studies with greater statistical power. Secondly, we did not do neutralization analysis against all DENV serotypes, which might help determine the history of infection and how it may have enhanced symptomatic infection due to prior DENV-2 or DENV-3 cross-reactive antibodies against DENV-4. Subjects from the current study were very likely exposed to DENV-3 (2009–2012), DENV-1 (2013–2014), and DENV-2 (2018–2021) [31,32,33,42]. Efforts to parse out preliminary exposure were performed by using a multiplex Luminex assay but increased response to several dengue serotypes in any given serum tested precluded any reliable conclusion (Supplement S7). Third, in dengue-vaccinated subjects infected with DENV-4, serum was not investigated for neutralization antibodies; thus, breakthrough infection was not confirmed, but future research is warranted.

## 5. Conclusions

In conclusion, we describe an epidemic of DENV-4 infection in patients presenting with AFI in León, Nicaragua. This outbreak was associated with a lineage of genotype GIIb that contained two amino acid changes not observed in DENV-4 before 2022. We found that pre-existing DENV antibody was frequent among those with acute DENV-4-related AFI; thus, further studies are needed to elucidate the immunological mechanism that might explain DENV-4 infection in seropositive subjects.

## Figures and Tables

**Table 1:** Epidemiological characteristics of febrile patients with and without acute DENV infection as determined by dengue RT-qPCR in Leon, Nicaragua, 2022.

Parameter	Total AFI Patients *n* = 172 (%)	Dengue RT-qPCR (+) *n* = 58 (%)	Dengue RT-qPCR (−) *n* = 114 (%)	*p*-Value	Prevalence Ratio (PR) (95%CI)
Sex					
Male	79 (46)	34 (59)	45 (40)	0.019	1.67 (1.09–2.56)
Residence					
Rural	32 (19)	12 (21)	20(17)		Ref
Urban	140 (81)	46 (79)	94 (83)	0.51	0.84 (0.51–1.39)
Age, median (IQR)	18 (10, 29)	15 (12, 22)	20 (8, 31)	0.28	N/A
Age group (years)					
≤11	48 (28)	12 (21)	36 (32)	0.95	1.03 (0.49–2.12)
12–18	43 (25)	25 (43)	18 (16)	0.004	2.38 (1.31–4.32)
19–29	40 (23)	11 (19)	29 (25)	0.75	1.13 (0.54–2.36)
≥30	41 (24)	10 (17)	31 (27)		Ref
Care given to the patient ^a^					
Ambulatory	130 (77)	36 (63)	94 (85)		Ref
Admitted to hospital	38 (23)	21 (37)	17 (15)	**0.001** ^b^	1.99 (1.34–2.97)
Reported contact with another ill individual within 7 days of illness	59 (34)	16 (28)	43 (38)	0.20	0.73 (0.45–1.18)
Asthma	3 (2)	1 (2)	2 (2)	0.99	0.99 (0.20–4.97)
Tuberculosis	3 (2)	2 (3)	1 (1)	0.09	2.01 (0.88–4.61)
Reported prior COVID-19					
No	130 (76)	41 (71)	89 (78)		Ref
Confirmed	16 (9)	3 (5)	13 (11)	0.33	0.56 (0.21–1.70)
Suspected	26 (15)	14 (24)	12 (11)	0.016 ^b^	1.71 (1.10–2.64)
Vaccination against					
COVID-19 ^c^	132 (78)	44 (77)	88 (78)	0.92	0.97 (0.59–1.61)
Dengue ^d^	5 (3)	2 (3)	3 (3)	0.74	1.21 (0.40–3.61)
Influenza ^c^	51 (30)	17 (30)	34 (30)	0.94	1.02 (0.64–1.62)
Pneumococcal ^d^	145 (85)	50 (86)	95 (84)	0.72	1.12 (0.60–2.08)
Haemophilus influenzae (Hib)	6 (4)	3 (5)	3 (3)	0.33	1.51 (0.66–3.46)
Antibiotics taken for this illness	17 (10)	3 (5)	14 (12)	0.19	0.49 (0.17–1.42)
Water sources					N/A
Tap/faucet	161 (94)	56 (97)	105 (92)		
Bottled	2 (1)	0	2 (2)		
Well	7 (4)	1 (2)	6 (5)		
Animal exposure					N/A
Pigs	11 (6)	0	11 (10)		
Cats	103 (60)	38 (66)	65 (57)		
Dogs	128 (74)	46 (79)	82 (72)		
Rodents	83 (48)	26 (45)	57 (50)		
Poultry	51 (30)	13 (22)	38 (33)		
Ticks	18 (11)	9 (16)	9 (8)		
Fleas	33 (19)	15(26)	18 (16)		
Mosquitoes	143 (83)	50 (86)	93 (82)		

^a^ *n* = 168; ^b^ after adjusting by serological status, no statistical differences were observed; ^c^ *n* = 170; ^d^ *n* = 171. To correct for multiple comparisons, we used a Bonferroni correction to set the type I error level at 0.05/18 comparisons. *p*-values less than 0.0028 are highlighted in bold to indicate statistical significance.

**Table 2:** Laboratory characteristics of febrile patients with and without RT-qPCR-confirmed acute DENV infection in Leon, Nicaragua, 2022.

**Laboratory Indicators** **(Acute)**	**RT-qPCR** **Positive *n* = 58** **Mean (±SD)**	**RT-qPCR** **Negative *n* = 113** **Mean (±SD)**	***p*-Value**
White blood cell count, 10^3^/µL ^g^	5.82 (2.59)	9.00 (7.68)	**0.003**
Eosinophil	1.56 (0.86)	3.63 (4.79)	0.004
Platelet count, 10^3^/µL ^j^	223 (617)	258 (660)	**0.001**
Neutrophil ^h^	65.05 (16.54)	62.28 (17.52)	0.34
Monocyte	8.92 (20.11)	3.29 (4.31)	0.36
Hemoglobin, g/mL ^b^	13.05 (1.37)	12.85 (1.59)	0.41
Lymphocyte ^i^	32.61 (16.08)	34.62 (16.30)	0.47
Mean corpuscular volume (MCV), fl ^k^	82.99 (5.18)	82.45 (9.15)	0.69
Hematocrit, %	38.10 (4.00)	38.35 (5.73)	0.74
Red blood cell count, 10^6^/µL ^b^	4.56 (4.86)	4.54 (6.99)	0.83
Basophil	2.67 (2.89)	3.38 (5.58)	0.84
**Laboratory Indicators** **(Convalescent) ^l^**	**RT-qPCR** **Positive *n* = 51** **Mean (±SD)**	**RT-qPCR** **Negative *n* = 86** **Mean (±SD)**	***p*-Value**
White blood cell count, 10^3^/µL ^g^	7.20 (2.18)	7.85 (2.40)	0.12
Eosinophil	3.09 (2.53)	3.59 (3.24)	0.44
Platelet count, 10^3^/µL ^g^	308 (9.14)	335(23.97)	0.35
Neutrophil	52.27 (11.74)	51.69 (11.40)	0.77
Monocyte	3.67 (2.55)	1.14 (0.38)	0.018
Hemoglobin, g/mL	13.01 (1.41)	12.69 (1.54)	0.22
Lymphocyte	44.76 (11.43)	44.84 (10.71)	0.97
Mean corpuscular volume (MCV), fl ^m^	80.49 (4.58)	79.75 (2.22)	0.39
Hematocrit, %	37.18 (4.02)	36.66 (4.76)	0.52
Red blood cell count, 10^6^/µL ^n^	4.59 (4.98)	4.65 (7.63)	0.62
Basophil	1.25 (0.46)	2.00 (2.19)	0.35

^b^ *n* = 170; ^g^ *n* = 169; ^h^ *n* = 156; ^i^ *n* = 161; ^j^ *n* = 165; ^k^ *n* = 155; ^l^ *n* = 137; ^m^ *n* = 136; ^n^ *n* = 133. To correct for multiple comparisons, we used a Bonferroni correction to set the type I error level at 0.05/11 comparisons for laboratory indicators (acute and convalescent). *p*-values less than 0.0045 are highlighted in bold to indicate statistical significance. Convalescent samples were collected at a mean of 30 days post symptom onset.

**Table 3:** Serologic profile of patients with acute DENV-4 infection among acute febrile illness in León, Nicaragua, 2022.

**Age Group (Years)**	**% of Patients with DENV IgG**	**DENV IgG**		**^c^ PR; 99%CI, *p***
		**Seropositive ^a^**	**Seronegative ^b^**	
		**% (RT-qPCR (+)/All Tested)**	**% (RT-qPCR (+)/All Tested)**	
≤11	50 (21/42)	38 (8/21)	5 (1/21)	8.38; 0.61–114.73; 0.036
12–18	87 (33/38)	61 (20/33)	40 (2/5)	1.52; 0.35–6.50; 0.462
19–29	89 (32/36)	25 (8/32)	25 (1/4)	1.00; 0.09–10.66; 1.000
≥30	97 (37/38)	19 (7/37)	100 (1/1)	N/A
All ages	79 (123/155)	35 (43/123)	16 (5/32)	2.24; 0.74–6.75; 0.060
**Sex**				
Male	81 (59/73)	49 (29/59)	7 (1/14)	6.88; 0.56–84.29; 0.047
Female	78 (64/82)	22 (14/64)	22 (4/18)	0.98; 0.27–3.57; 0.975
Both	79 (123/155)	35 (43/123)	16 (5/32)	2.24; 0.74–6.75; 0.060
**Age Group (Years)**	**% of Patients with Zika IgG**	**Zika IgG ^d^**		**PR; 99%CI, *p***
		**Seropositive**	**Seronegative**	
		**% (RT-qPCR (+)/All Tested)**	**% (RT-qPCR (+)/All Tested)**	
≤11	13 (5/40)	60 (3/5)	14 (5/35)	4.20; 1.01–17.41; 0.009
12–18	51 (19/37)	58 (11/19)	61 (11/18)	0.95; 0.47–1.90; 0.842
19–29	49 (17/35)	18 (3/17)	33 (6/18)	0.53; 0.11–2.62; 0.306
≥30	39 (14/36)	7 (1/14)	32 (7/22)	0.22; 0.02–3.05; 0.14
All ages	37 (55/148)	33 (18/55)	31 (29/93)	1.05; 0.56–1.98; 0.85
**Sex**				
Male	42 (30/71)	47 (14/30)	37 (15/41)	1.28; 0.62–2.65; 0.391
Female	32 (25/77)	16 (4/25)	27 (14/52)	0.59; 0.16–2.22; 0.309
Both	37 (55/148)	33 (18/55)	31 (29/93)	1.05; 0.56–1.98; 0.85

^a^ If the acute serum was both DENV IgG-positive and RT-qPCR-positive, the patient was experiencing a secondary infection. ^b^ If the acute serum was both DENV IgG-negative and RT-qPCR-positive, the patient was experiencing a primary infection. ^c^ We used generalized linear models to estimate the prevalence ratios (PRs). ^d^ Seven samples were excluded. To correct for multiple comparisons, we used a Bonferroni correction to set the type I error level at 0.05/5 comparisons for age groups (DENV IgG) and the CI level at (1–0.05/5). To correct for multiple comparisons, we used a Bonferroni correction to set the type I error level at 0.05/6 comparisons for age groups (Zika IgG).

## Data Availability

All raw data associated with the figures are either included in this submission or are available upon request to the corresponding authors. The sequences for DENV-4 have been deposited in GenBank under the accession numbers GenBank PP128470–PP128485.

## Supplementary Materials

The following supporting information can be downloaded at: https://www.mdpi.com/article/10.3390/v17081113/s1. Supplement S1. Extended and detailed Material and Methods. Supplement S2. DENV-4 virus (*n* = 15) and DENV-1 virus (*n* = 2) were successfully isolated from acute samples by three serial passages of serum on Vero cells. Serum was diluted 1/10 in isolation medium (2% FBS-DMEM). RT-PCR was performed before and after serial culture, with decreases in Ct values in post culture testing indicating replication of the viral isolate. Genomic sequencing was performed for 7 of the isolates and % genome coverage is indicated in the final column. Supplement S3. Clinical manifestations and vital signs parameters of febrile patients with acute DENV infection as determined by dengue RT-qPCR in León, Nicaragua 2022. Supplement S4. Sensitive analysis by including the 6 samples that were treated as false positives. Supplement S5. Alignment of the amino acid sequences of the E protein from the DENV-4 Nicaraguan 2022 variant and several references strains. Supplement S6. Statistical analysis without the Bonferroni correction. Supplement S7. Multiplex Luminex assay to determine serotype specific response in a subset of subjects and to emphasize that in dengue endemic areas multiple exposures or serotype cross reactive antibodies ruled out the possibility to determine history of infection. The same challenge is experienced by using neutralization assays.
