# Supplementary material for: Acute Febrile Illness Associated with an Emerging Dengue 4 GIIb Variant Causing Epidemic in León, Nicaragua 2022
Source: Viruses. 2025 Aug 13;17(8):1113. doi: 10.3390/v17081113 (PMC12390644; doi:10.3390/v17081113)
Supplement: Supplementary file 1 [file viruses-17-01113-s001.zip › viruses-3738178-final supplementary.pdf]

Supplement 1

MATERIAL AND METHODS

Nicaraguan acute febrile illness (AFI) cohort and samples. The hospital and community surveillance cohort for AFI established in western (León) Nicaragua since 2017 detected a sharp increase in febrile cases in June 2022. The current study includes adults and pediatric patients recruited between June 1st and September 30th, 2022, at the emergency department of the main local hospital and two community clinics. Those patients of ≥ 1 years of age with documented fever (≥38°C, tympanic) within 48 hours of enrollment were eligible for inclusion. We excluded patients with focal bacterial infections and recent (≤ 7 days) trauma or hospitalization. Patients provided acute- and convalescent-phase samples. Within 3 hours of venipuncture, blood was processed, sera aliquoted, and specimens stored at -80°C. A separate aliquot not previously thawed was shipped to Emory University on dry ice for dengue serology, virus isolation, and sequencing analysis.

Ethics statement. Written informed consent was obtained from patients or their guardians (for patients ≤18 years of age), and written assent was obtained from patients aged 12 -17 years. The study protocol was reviewed and approved by the institutional review boards of UNAN-León (FWA00004523/IRB00003342) and Duke University (Pro00014461). The with de-identified specimens was declared exempt from IRB review by Emory University.

Arbovirus screening and dengue serotyping. RNA was purified from 140µl of serum using the QIAamp Viral RNA Mini Kit (QIAmp, Hilden, Germany). Viral RNA reverse transcription (RT) and quantitative polymerase chain reaction (qPCR) was performed in real time by using the CFX96 equipment (Bio-Rad, Hercules, California, USA), primers and probes

included in Trioplex assay[1], and the AgPath-ID™ One-Step RT-qPCR Reagents (Applied Biosystem, Waltham, Massachusetts). Any sample showing exponential amplification with either FAM, HEX or Fluor Red 61 with Ct value <38 was considered positive for either Dengue, Chikungunya or Zika virus, respectively. Amplification of the human RNase P gene confirmed successful RNA extraction. The positive-control was RNA from inactivated viruses and the non-template control was PCR-grade water. All RT-qPCR positive and a subset of randomly selected DENV-negative samples were re-tested at Emory University to define serotypes and to augment rigor of molecular testing results. These procedures for a triplex (DENV, ZIKV and CHIKV) and DENV serotyping RT-PCR assays are described elsewhere [2],[3].

Name	Primer Sequence (5'→3')	Genomic Location
DENV-1, 2, 3 Forward	*CAGATCTCTGATGAACAACCAACG*	86–109
DENV-2 Forward C→T	*CAGATCTCTGATGAATAACCAACG*	87–110
DENV-3 Forward C→T	*CAGATTTCTGATGAACAACCAACG*	85–108
DENV-4 Forward	*GATCTCTGGAAAAATGAAC*	81–99
DENV-1, 3 Reverse	*TTTGAGAATCTCTTCGCCAAC*	DENV-1: 199-179, DENV-3: 198-178
DENV-2 Reverse	*AGTTGACACGCGGTTTCTCT*	171-152
DENV-2 Reverse A→G	*AGTCGACACGCGGTTTCTCT*	171-152
DENV-4 Reverse	*AGAATCTCTTCACCAACC*	190-173

Genomic locations are provided for the 5′ base of each primer based on reference virus sequences: DENV-1, US/Hawaii/1944 (GenBank: EU848545.1); DENV-2, New Guinea C Strain (GenBank: AF038403.1); DENV-3, strain H87 (GenBank: M93130.1); DENV-4, strain H241 (GenBank: AY947539.1). Predicted amplicon sizes are as follows: DENV-1, 114 bp; DENV-2, 85 bp; DENV-3, 114 bp; DENV-4, 110 bp.

Dengue genome sequencing and phylogenetic analysis. A subset (n=16) of samples with Ct values ≤ 32 were selected for full genome sequencing. Total nucleic acid was treated with

dsDNase (ArcticZymes, Tromso, Norway). RNA was converted into cDNA using random hexamer primers and SuperScript III RT (Fisher/Invitrogen) for first strand synthesis and New England Biolabs reagents for second strand synthesis without amplification. Libraries were fragmented, dual indexed, and amplified with 16 PCR cycles using the Nextera XT DNA Library Prep kit (Illumina). Libraries were quantified using the KAPA universal complete kit (Roche), pooled to equimolar concentration, and sequenced on a MiSeq with paired-end 150-bp reads (Illumina) with a mean of 935,280 reads per sample (range 502,300 - 1,560,818). As a negative control, water was included with each batch of samples starting from DNase. As a positive control, in vitro transcribed ERCC spike-ins (NIST) were added to each sample prior to cDNA synthesis.

Reads underwent reference-based DENV genome assembly by using as DENV-4 reference the EU854296 genome and the viral-ngs version 2.0.21.3 (https://github.com/broadinstitute/viral-ngs). Dengue serotype and genotype were determined using the Dengue Virus Typing Tool[4]. Sequences of the Nicaraguan 2022 isolates were then aligned with 280 reference sequences, which were selected as belonging to DENV-4, genotype II; including complete genome sequences (>94% coverage); and having known date and location of sampling. A maximum-likelihood circular tree was constructed from the full coding region using a GTR+F+I+G4 model (best fit) in IQ-TREE version 2.0[5]. Trees were visualized using iTOL[6]. Pairwise distance (PD) calculation was performed to determine degree of homology between the Envelope (E) protein from Nicaraguan 2022 isolates and the E protein from all know references DENV-4 strains, including vaccines strains. Phylogenetic analysis was performed using the MEGA 11.0.13 and the tree was constructed using the neighbor-joining and Kimura two-parameter methods.

Antigen capture IgG ELISA. Binding IgG to DENV was measured by antigen capture ELISA as previously described[7]. Briefly, wells of a 96-well ELISA plate were coated at room

temperature with 50 uL of 2ug/mL of the anti-E protein mouse mAb 4G2 in 0.1M Carbonate Buffer (pH 9.6), then blocked with 3% nonfat dry milk in PBS-T overnight at 4°C. Plates were washed with 0.05% Tween-20-PBS and incubated with DENV antigens (an equal volume mixture of supernatant from each of the four DENV serotypes cultured in C6/36 cells). After washing, serum was incubated at the desired dilution at 37°C for 1 hour. DENV-binding IgG was detected with an alkaline phosphatase-conjugated goat anti-human IgG and p-nitrophenyl phosphate substrate. Optical density (OD) was measured at 405 nm by spectrophotometry. ELISA data were reported as OD values that were the average of technical replicates. Serum from flavivirus-naïve individuals served as the negative control (NC) in each ELISA assay. The cut off for positivity in each plate was 0.1 plus the average OD of the NCs plus 3 standard deviations.

Supplement 2.

DENV-4 virus (n=15) and DENV-1 virus (n=2) were successfully isolated from acute samples by three serial passages of serum on Vero cells. Serum was diluted 1/10 in isolation medium (2% FBS-DMEM). RT-PCR was performed before and after serial culture, with decreases in Ct values in post culture testing indicating replication of the viral isolate. Genomic sequencing was performed for 7 of the isolates and % genome coverage is indicated in the final column.

	Ct Pre-Culture					Ct post- culture					% of Sequence coverage
	**DENV-1**	**DENV-2**	**DENV-3**	**DENV-4**	**Resultado Final**	**DENV-1**	**DENV-2**	**DENV-3**	**DENV-4**	**Resultado Final**	
NIC-1	N	N	N	24.58	Pos	N	N	N	N	N	100
NIC-2	N	N	N	18.57	Pos	N	N	N	13.13	D4	100
NIC-3	N	N	N	22.81	Pos	N	N	N	N	N	100
NIC-4	N	N	N	N	Neg	N	N	N	N	N	
NIC-5	N	N	N	28.62	Pos	N	N	N	N	N	100
NIC-6	N	N	N	22.28	Pos	N	N	N	12.26	D4	100
NIC-7	N	N	N	28.09	Pos	N	N	N	13.15	D4	
NIC-8	N	N	N	24.67	Pos	N	N	N	13.41	D4	
NIC-9	N	N	N	22.24	Pos	N	N	N	12.79	D4	
NIC-10	N	N	N	20.29	Pos	N	N	N	13.05	D4	100
NIC-11	N	N	N	20.56	Pos	N	N	N	13.4	D4	100
NIC-12	N	N	N	22.86	Pos	N	N	N	N	N	
NIC-13	N	N	N	23.27	Pos	N	N	N	N	N	
NIC-14	N	N	N	22.69	Pos	35.55	N	N	38.26		
NIC-15	21.98	N	N	19.99	Pos- D1	13.85	N	N	14.94	D1	
NIC-16	N	N	N	28.5	Pos	N	N	N	N	N	
NIC-17	N	N	N	22.89	Pos	N	N	N	N	N	100
NIC-18	N	N	N	N	Neg	N	N	N	N	N	
NIC-19	N	N	N	25.6	Pos	N	N	N	N	N	
NIC-20	N	N	N	23.22	Pos	N	N	N	N	N	100
NIC-21	N	N	N	29.38	Pos	N	N	N	N	N	
NIC-22	N	N	N	23.68	Pos	N	N	N	13.14	D4	
NIC-23	N	N	N	30.42	Pos	N	N	N	N	N	
NIC-24	N	N	N	20.46	Pos	N	N	N	13.08	D4	
NIC-25	N	N	N	27.66	Pos	N	N	N	13.58	D4	
NIC-26	N	N	N	30.83	Pos	N	N	N	N	N	unsuccessful
NIC-27	N	N	N	28.46	Pos	N	N	N	N	N	99
NIC-28	N	N	N	22.54	Pos	N	N	N	N	N	
NIC-29	N	N	N	23.95	Pos	N	N	N	N	N	100
NIC-30	N	N	N	21.12	Pos	N	N	N	12.58	D4	100
NIC-31	N	N	N	23.03	Pos	N	N	N	12.98	D4	
NIC-32	N	N	N	25.61	Pos	N	N	N	12.68	D4	100
NIC-33	N	N	N	23.21	Pos	N	N	N	N	N	
NIC-34	N	N	N	N	Neg	N	N	N	N	N	
NIC-35	N	N	N	23.13	Pos	N	N	N	14.9	D4	100
NIC-36	N	N	N	30.33	Pos	N	N	N	N	N	
NIC-37	20.32	N	N	18.78	Pos- D1	13.25	N	N	13.15	D1	100
NIC-38	N	N	N	32.99	Pos	N	N	N	N	N	unsuccessful
NIC-39	N	N	N	25.52	Pos	N	N	N	N	N	100
NIC-40	N	N	N	20.85	Pos	N	N	N	12.03	D4	
NIC-41	N	N	N	30.36	Pos	N	N	N	N	N	
NIC-42	N	N	N	N	Neg						
NIC-43	N	N	N	33.62	Pos	N	N	N	N	N	
NIC-44	N	N	N	N	Neg						
NIC-45	N	N	N	30.18	Pos	N	N	N	N	N	unsuccessful
NIC-46	N	N	N	23.61	Pos	N	N	N	N	N	100
NIC-47	N	N	N	22.95	Pos	N	N	N	N	N	
NIC-48	N	N	N	25.16	Pos	N	N	N	N	N	

Supplement 3.

Clinical manifestations and vital signs parameters of febrile patients with acute DENV infection as determined by dengue RT-qPCR in León, Nicaragua 2022.

Clinical Manifestations	Dengue RT-qPCR Trioplex AFI patients N=172			
	**Positive n= 58 (%)**	**Negative n= 114 (%)**	***p*-value**	**Prevalence Ratio (PR) (95%CI)**
Fever	58 (100)	114 (100)	-	-
Headache	53 (91)	84 (74)	0.020	2.71 (1.17-6.26)
Rash	10 (18)	7 (6)	0.006	1.93 (1.21-3.06)
Joint pain	40 (69)	57 (50)	0.023	1.72 (1.08-2.74)
Retroorbital pain	28 (49)	37 (33)	0.037	1.56 (1.03-2.37)
Muscle pain	40 (69)	61 (54)	0.06	1.56 (0.98-2.49)
Fatigue[c]	9 (16)	11 (10)	0.23	1.39 (0.81-2.37)
Chills	49 (85)	91 (80)	0.47	1.24 (0.68-2.26)
Dysuria	6 (10)	9 (8)	0.57	1.21 (0.63-2.33)
Abdominal pain	20 (35)	34 (30)	0.53	1.15 (0.74-1.78)
Loss of taste	2 (4)	4 (4)	0.99	0.99 (0.31-3.15)
Diminished urination	6 (10)	13 (11)	0.84	0.93 (0.46-1.87)
Epistaxis	1 (2)	3 (3)	0.73	0.74 (0.13-4.10)
Vomiting	8 (14)	27 (24)	0.16	0.63 (0.33-1.19)
Conjunctivitis	1 (2)	4 (4)	0.56	0.59 (0.10-3.45)
Dyspnea[c]	3 (5)	15 (13)	0.15	0.46 (0.16-1.33)
Sore throat	9 (16)	50 (44)	**0.001**	0.35 (0.19-0.67)
Diarrhea	3 (5)	26 (23)	0.018	0.27 (0.09-0.80)
Rhinorrhea	7 (12)	61 (54)	**0.000**	0.21 (0.10-0.44)
Cough[d]	6 (10)	59 (52)	**0.000**	0.19 (0.09-0.42)
**Vital signs (Acute)**		**RT- qPCR Positive n= 58 Mean (±SD)**	**RT-qPCR Negative n= 114 Mean (±SD)**	**p – value**
Documented Temperature (˚C)[e]		38.72 (0.74)	38.42 (0.70)	0.029
Actual Temperature (˚C)		37.49 (2.22)	37.53 (5.25)	0.96
Systole blood pressure (mmHg)[f]		109.02(12.81)	104.14 (14.82)	0.037
Diastole blood pressure (mmHg)[f]		66.84 (8.34)	67.40 (9.45)	0.71
O_2 Saturation (%)[b]		97.95 (1.08)	97.35 (3.05)	0.15
Respiratory rate (/min)[c]		19.69 (2.04)	19.54 (2.05)	0.65
Heart rate (beats/min)[c]		96.46 (15.34)	94.37 (22.43)	0.53

[b]n=170, [c]n=171, [d]n=144 and included dry cough and productive cough, [e]n=128, [f]n=161. To correct for multiple comparisons, we use a Bonferroni correction to set the type I error level at 0.05/19 comparisons for clinical manifestations. *P* – value less than 0.0026 are given highlighted in bold to indicate statistical significance. To correct for multiple comparisons, we use a Bonferroni correction to set the type I error level

at 0.05/7 comparisons for vital signs. P – value less than 0.007 are given highlighted in bold to indicate statistical significance.

Supplement 4.

Sensitive analysis by including the 6 samples that were treated as false positives. Geometric mean (GM) and mean plots with 95% confidence intervals (CI) comparing A) WBC, B) platelet, and C) eosinophil counts across acute and no acute. D) WBC and E) Platelets counts examined in blood collected from secondary dengue during acute and convalescence phases.

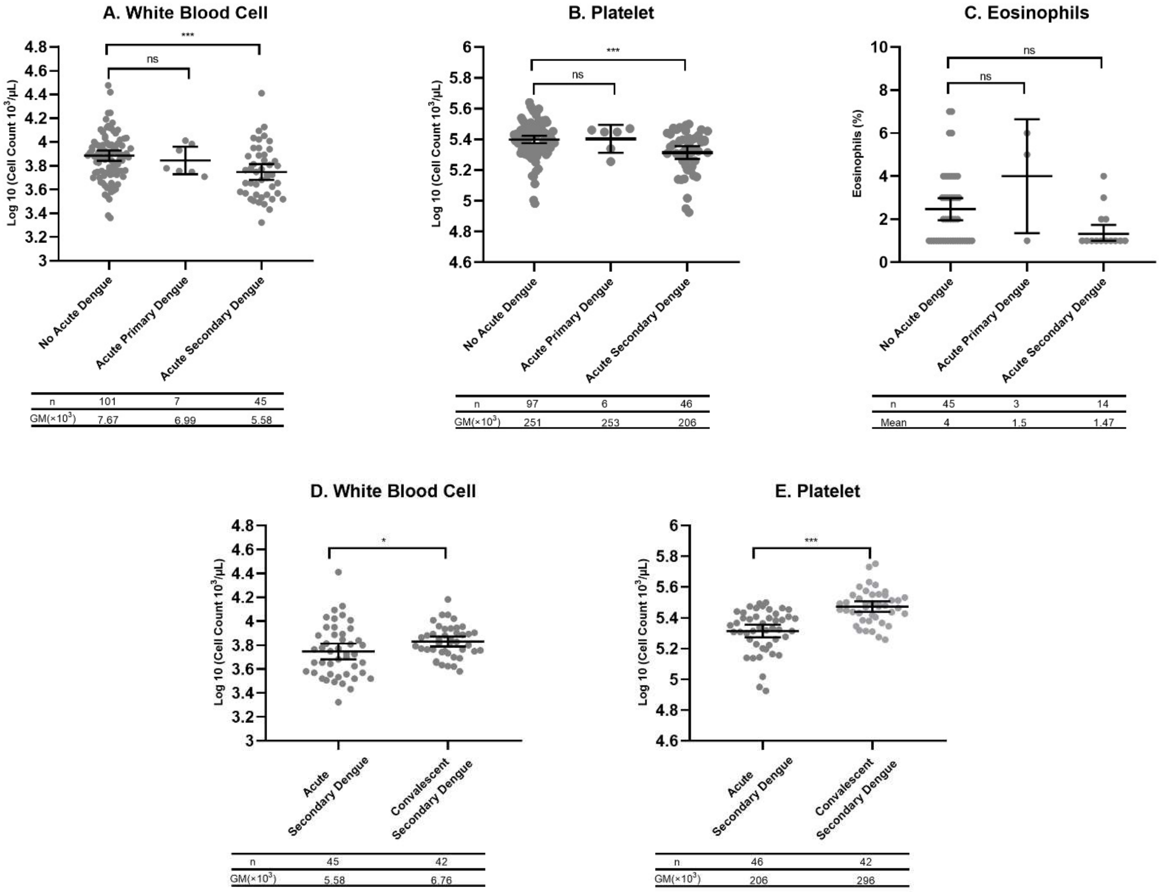

Supplement 5.

Aminoacids alignment of the E protein from the DENV-4 Nicaraguan 2022 variant and several references strains.

Nica-DENV-001A	MRCVGVGNRDFVEGVSGGAWVDLVLEHGGCVTTMAQGKPTLDFELTKTTAKEVALLRTYCIEASISNITTATRCPTQGEPYLKEEQDQQYICRRDVVDRGWGNGCGLFGKGGVVTCAKFSCSGK
Nica-DENV-003C	....
Nica-DENV-004D	....
Nica-DENV-007G	....
Nica-DENV-008H	....
Nica-DENV-009I	....
Nica-DENV-011K	....
Nica-DENV-015O	....
Nica-DENV-016P	....
Nica-DENV-014N	....
Nica-DENV-010J	....
Nica-DENV-017Q	....
Nica-DENV-012L	....
Nica-DENV-006F	...I..
Nica-DENV-013M	....
Nica-DENV-005E	....
OQ821646 El Salvador 2022	....V..
OM417342 ChiapasMexico Oct2021	....
OQ445967 FloridaUSA 2022	....
KU513442 TVP/360 Brazil 2016	....
KT452803 Nicaragua1999	....
AF326573 Dominica 1981	....
GIIb FJ882599 PuertoRico 1999	....
GIIb KJ160504 GII SriLanka 1992	....
GIIa JN832541 FrenchPolynesia2009	....L....
GI KF543272 GI Cambodia 2010	....I...M.........................L..
GIII AY618940 Thailand 2001	...I...I..
GIV AF231724 Malasya 1973	..................T..........................I..
GV JF262783 INDIA 1961	....I.......T...L....
U18429 Indonesia1976 TAK-003	....
KX239897 YD4 1228 TDV-980 Deng	....L....

Nica-DENV-001A	ITGNLVQIENLEYTVVVTVHNGDTHAVGNDTSNHGVTATITPRSPSVGVNLPDYGELTLDCEPRSGIDFNEMILMKMKKKTWLVHKQWFLDLPLPWTAGADTSEVHWNHKERMVTFKVPHAKRQ
Nica-DENV-003C	....
Nica-DENV-004D	....
Nica-DENV-007G	....
Nica-DENV-008H	....
Nica-DENV-009I	....
Nica-DENV-011K	....
Nica-DENV-015O	....
Nica-DENV-016P	....
Nica-DENV-014N	....
Nica-DENV-010J	....
Nica-DENV-017Q	....
Nica-DENV-012L	....
Nica-DENV-006F	....
Nica-DENV-013M	....
Nica-DENV-005E	....
OQ821646 El Salvador 2022	....
OM417342 ChiapasMexico Oct2021	....
OQ445967 FloridaUSA 2022	....
KU513442 TVP/360 Brazil 2016	......................................M........E.K..Y...............
KT452803 Nicaragua1999	....E.K..Y...............
AF326573 Dominica 1981	......................................M........E.K..Y...............
GIIb FJ882599 PuertoRico 1999	.....................................T.........E.K..Y...............
GIIb KJ160504 GII SriLanka 199	....E.K..Y...............
GIIa JN832541 FrenchPolynesia	....E.K..Y...............
GI KF543272 GI Cambodia 2010	....E.K............................T..................T....L.....................
GIII AY618940 Thailand 2001	....E.E.......S...................E..
GIV AF231724 Malasya 1973	.......V...............A.....S.......T.........E.K............................G.............................Y...............
GV JF262783 INDIA 1961	.........................V..K..................E.K...Q..Y...............
U18429 Indonesia1976 TAK-003	....E.K..Y...............
KX239897 YD4 1228 TDV-980 Deng	....E.K..Y...............

Nica-DENV-001A	DVTVLGSQEGAMHSALAGATEVDSGDGNHMFAGHLKCKVRMEKLRIKGMSYTMCSGKFSIDKEMAETQHGTTVVKVKYEGAGAPCKVPIEIRDVNKEKVVGRVISSTPLAENTNSVTNIELEPP
Nica-DENV-003C	....
Nica-DENV-004D	....
Nica-DENV-007G	....
Nica-DENV-008H	....
Nica-DENV-009I	....
Nica-DENV-011K	....
Nica-DENV-015O	....
Nica-DENV-016P	....
Nica-DENV-014N	....
Nica-DENV-010J	.............................I..
Nica-DENV-017Q	....
Nica-DENV-012L	....
Nica-DENV-006F	....
Nica-DENV-013M	....
Nica-DENV-005E	....
OQ821646 El Salvador 2022	....
OM417342 ChiapasMexico Oct2021	....C.........
OQ445967 FloridaUSA 2022	....
KU513442 TVP/360 Brazil 2016	....I.....................
KT452803 Nicaragua1999	....
AF326573 Dominica 1981	....I.....................
GIIb FJ882599 PuertoRico 1999	....
GIIb KJ160504 GII SriLanka 199	....
GIIa JN832541 FrenchPolynesia	....I...............I.....F.......I.......
GI KF543272 GI Cambodia 2010	...........V..T.....................I.....F...............
GIII AY618940 Thailand 2001	................T..R..................T.....................I.....F...............
GIV AF231724 Malasya 1973	................T...T.....I....K.M........I...I.F......I........
GV JF262783 INDIA 1961	................T...I.....F...............
U18429 Indonesia1976 TAK-003	....P...I............A........
KX239897 YD4 1228 TDV-980 Deng	....I.....F...............

Nica-DENV-001A	FGDSYIVIGVGNSALTLHWFRKGSSIGKMFESTYRGAKRMAILGETAWDFGSVGGLFTSLGKAVHQVFGSVYTTMFGGVSWMIRILIGFLVLWIGTNSRNTSMAMTCIAVGGITLFLGFTVQA-
Nica-DENV-003C	....-
Nica-DENV-004D	....-
Nica-DENV-007G	....-
Nica-DENV-008H	....-
Nica-DENV-009I	....-
Nica-DENV-011K	....-
Nica-DENV-015O	....-
Nica-DENV-016P	.....X...-
Nica-DENV-014N	......M..-
Nica-DENV-010J	....-
Nica-DENV-017Q	....-
Nica-DENV-012L	....-
Nica-DENV-006F	....-
Nica-DENV-013M	....V..-
Nica-DENV-005E	....V..-
OQ821646 El Salvador 2022	....-
OM417342 ChiapasMexico Oct2021	....-
OQ445967 FloridaUSA 2022	....X-------------------------
KU513442 TVP/360 Brazil 2016	....X-------------------------
KT452803 Nicaragua1999	....-
AF326573 Dominica 1981	....-
GIIb FJ882599 PuertoRico 1999	....-
GIIb KJ160504 GII SriLanka 199	....-
GIIa JN832541 FrenchPolynesia	...........D...-
GI KF543272 GI Cambodia 2010	...........DG...V.....L................S...............H.X
GIII AY618940 Thailand 2001	...........D..V.....L............P.....................-
GIV AF231724 Malasya 1973	.........A.D...I................S.................-
GV JF262783 INDIA 1961	...........D..V.....L....................I...........H.-
U18429 Indonesia1976 TAK-003	....L..-
KX239897 YD4 1228 TDV-980 Deng	...........D...M..........................V......................................G.-

Supplement 6.

Statistical analysis without the Bonferroni correction

Clinical manifestations and vital signs parameters of febrile patients with acute DENV infection as determined by dengue RT-PCR in León, Nicaragua 2022.

Clinical Manifestations	Dengue RT-qPCR Trioplex			
	Acute N=172			
	**Positive n= 58 (%)**	**Negative n= 114 (%)**	***p*-value**	**Prevalence Ratio (PR) (95%CI)**
Fever	58 (100)	114 (100)	-	-
Headache	53 (91)	84 (74)	**0.020**	2.71 (1.17-6.26)
Rash	10 (18)	7 (6)	**0.006**	1.93 (1.21-3.06)
Joint pain	40 (69)	57 (50)	**0.023**	1.72 (1.08-2.74)
Retroorbital pain	28 (49)	37 (33)	**0.037**	1.56 (1.03-2.37)
Muscle pain	40 (69)	61 (54)	0.06	1.56 (0.98-2.49)
Fatigue[c]	9 (16)	11 (10)	0.23	1.39 (0.81-2.37)
Chills	49 (85)	91 (80)	0.47	1.24 (0.68-2.26)
Dysuria	6 (10)	9 (8)	0.57	1.21 (0.63-2.33)
Abdominal pain	20 (35)	34 (30)	0.53	1.15 (0.74-1.78)
Loss of taste	2 (4)	4 (4)	0.99	0.99 (0.31-3.15)
Diminished urination	6 (10)	13 (11)	0.84	0.93 (0.46-1.87)
Epistaxis	1 (2)	3 (3)	0.73	0.74 (0.13-4.10)
Vomiting	8 (14)	27 (24)	0.16	0.63 (0.33-1.19)
Conjunctivitis	1 (2)	4 (4)	0.56	0.59 (0.10-3.45)
Dyspnea[c]	3 (5)	15 (13)	0.15	0.46 (0.16-1.33)
Sore throat	9 (16)	50 (44)	**0.001**	0.35 (0.19-0.67)
Diarrhea	3 (5)	26 (23)	**0.018**	0.27 (0.09-0.80)
Rhinorrhea	7 (12)	61 (54)	**0.000**	0.21 (0.10-0.44)
Cough[d]	6 (10)	59 (52)	**0.000**	0.19 (0.09-0.42)

**Vital signs (Acute)**	**RT- qPCR Positive n= 58 Mean (±SD)**	**RT-qPCR Negative n= 114 Mean (±SD)**	***p* – value**
Documented Temperature (˚C)[e]	38.72 (0.74)	38.42 (0.70)	**0.029**
Actual Temperature (˚C)	37.49 (2.22)	37.53 (5.25)	0.96
Systole blood pressure (mmHg)[f]	109.02(12.81)	104.14 (14.82)	**0.037**
Diastole blood pressure (mmHg)[f]	66.84 (8.34)	67.40 (9.45)	0.71
O_2 Saturation (%)[b]	97.95 (1.08)	97.35 (3.05)	0.15
Respiratory rate (/min)[c]	19.69 (2.04)	19.54 (2.05)	0.65
Heart rate (beats/min)[c]	96.46 (15.34)	94.37 (22.43)	0.53

[b]n=170, [c]n=171, [d]n=144 and included dry cough and productive cough, [e]n=128, [f]n=161. *P* – value less than 0.05 are given highlighted in bold to indicate statistical significance.

Supplement 7.

Multiplex Luminex assay to determine serotype specific response. Multiplex Luminex assay to determine serotype specific response in a subset of subjects and to emphasize that in dengue endemic areas multiple exposures or serotype cross reactive antibodies ruled out the possibility to determine history of infection. The same challenge is experienced by using neutralization assays. The multiplex serological assay is a microsphere-based serological assay using domain III of the envelope protein (EDIII) of DENV serotypes 1–4 and Zika, the most variable region between each virus[8].

	S. NO	Dilution	DV1 EDIII Signal Cut-Off= 1306	DV2 EDIII Signal Cut-Off= 1082	DV3 EDIII Signal Cut-Off= 1364	DV4 EDIII Signal Cut-Off= 1285	Zika EDIII Signal Cut-Off= 987	Anti IgG	Halo Tag
**1-1761-JBT**	DV4-46	1to500	2862	20320	5455	19376	18451	17112	2360
**1-1745-BQR**	DV4-40	1to500	9444	19909	19204	18775	18906	17744	945
**1-1777-JGF**	DV4-48	1to500	13534	9830	18882	10302	8462	17205	619
**1-1771-LPI**	DV4-47	1to500	3169	20790	6254	3986	933	18198	516
**1-1744-JAM**	DV4-39	1to500	93	62	66	73	110	17278	67

REFERENCES

1. Santiago GA, Vázquez J, Courtney S, et al. Performance of the Trioplex real-time RT-PCR assay for detection of Zika, dengue, and chikungunya viruses. *Nat Commun*. 2018;9(1):1391. doi:10.1038/s41467-018-03772-1

2. Waggoner JJ, Abeynayake J, Sahoo MK, et al. Single-Reaction, Multiplex, Real-Time RT-PCR for the Detection, Quantitation, and Serotyping of Dengue Viruses. Morrison AC, ed. *PLoS Negl Trop Dis*. 2013;7(4):e2116. doi:10.1371/journal.pntd.0002116

3. Waggoner JJ, Gresh L, Mohamed-Hadley A, et al. Single-Reaction Multiplex Reverse Transcription PCR for Detection of Zika, Chikungunya, and Dengue Viruses. *Emerg Infect Dis*. 2016;22(7):1295-1297. doi:10.3201/eid2207.160326

4. Vilsker M, Moosa Y, Nooij S, et al. Genome Detective: an automated system for virus identification from high-throughput sequencing data. Birol I, ed. *Bioinformatics*. 2019;35(5):871-873. doi:10.1093/bioinformatics/bty695

5. Nguyen LT, Schmidt HA, von Haeseler A, Minh BQ. IQ-TREE: a fast and effective stochastic algorithm for estimating maximum-likelihood phylogenies. *Mol Biol Evol*. 2015;32(1):268-274. doi:10.1093/molbev/msu300

6. Letunic I, Bork P. Interactive Tree Of Life (iTOL) v4: recent updates and new developments. *Nucleic Acids Research*. 2019;47(W1):W256-W259. doi:10.1093/nar/gkz239

7. Collins MH, Tu HA, Gimblet-Ochieng C, et al. Human antibody response to Zika targets type-specific quaternary structure epitopes. *JCI Insight*. 2019;4(8):e124588, 124588. doi:10.1172/jci.insight.124588

8. Hein LD, Castillo IN, Medina FA, et al. Multiplex sample-sparing assay for detecting type-specific antibodies to Zika and dengue viruses: an assay development and validation study. *Lancet Microbe*. 2025;6(2):100951. doi:10.1016/j.lanmic.2024.07.014
